# In Vitro Evaluation of Chemical and Microhardness Alterations in Human Enamel Induced by Three Commercial In-Office Bleaching Agents

**DOI:** 10.3390/dj13080357

**Published:** 2025-08-06

**Authors:** Berivan Laura Rebeca Buzatu, Atena Galuscan, Ramona Dumitrescu, Roxana Buzatu, Magda Mihaela Luca, Octavia Balean, Gabriela Vlase, Titus Vlase, Iasmina-Mădălina Anghel, Carmen Opris, Bianca Ioana Todor, Mihaela Adina Dumitrache, Daniela Jumanca

**Affiliations:** 1 Translational and Experimental Clinical Research Centre in Oral Health, Department of Preventive, Community Dentistry and Oral Health, “Victor Babes” University of Medicine and Pharmacy, 300040 Timisoara, Romania; berivan.buzatu@umft.ro (B.L.R.B.); galuscan.atena@umft.ro (A.G.); balean.octavia@umft.ro (O.B.); jumanca.daniela@umft.ro (D.J.); 2 Clinic of Preventive, Community Dentistry and Oral Health, “Victor Babes” University of Medicine and Pharmacy, Eftimie Murgu Sq. no 2, 300041 Timisoara, Romania; 3 Department of Dental Aesthetics, Faculty of Dental Medicine, “Victor Babes” University of Medicine and Pharmacy Timisoara, Revolutiei Boulevard 9, 300041 Timisoara, Romania; roxana.buzatu@umft.ro; 4 Department of Pediatric Dentistry, Faculty of Dental Medicine, “Victor Babes” University of Medicine and Pharmacy Timisoara, Eftimie Murgu Square 2, 300041 Timisoara, Romania; luca.magda@umft.ro; 5 Research Centre for Thermal Analysis in Environmental Problems-ICAM, West University of Timisoara, Pestalozzi Street 16, 300115 Timisoara, Romania; gabriela.vlase@e-uvt.ro (G.V.); titus.vlase@e-uvt.ro (T.V.); 6 Faculty of Mechanics, Department of Materials Engineering and Manufacturing, Politehnica University, 300222 Timisoara, Romania; iasmina.anghel@student.upt.ro (I.-M.A.); carmen.opris@upt.ro (C.O.); 7 Department of Dentistry, Faculty of Medicine and Pharmacy, University of Oradea, 410087 Oradea, Romania; btodor@uoradea.ro; 8 Oral Health and Community Dentistry Department, Faculty of Dental Medicine, Carol Davila University of Medicine and Pharmacy, 020021 Bucharest, Romania

**Keywords:** tooth bleaching, Vickers microhardness, FTIR spectroscopy, hydrogen peroxide, carbamide peroxide

## Abstract

**Background/Objectives**: In-office bleaching commonly employs high concentrations of hydrogen peroxide (HP) or carbamide peroxide (CP), which may compromise enamel integrity. This in vitro paired-design study aimed to compare the chemical and mechanical effects of three commercial bleaching agents—Opalescence Boost (40% HP), Opalescence Quick (45% CP), and BlancOne Ultra+ (35% HP)—on human enamel. The null hypothesis assumed no significant differences between the control and treated samples. Given the ongoing debate over pH, active ingredients, and enamel impact, comparing whitening systems remains clinically important. **Methods**: Forty-two extracted teeth were assigned to three experimental groups (*n* = 14) with matched controls. Each underwent a single bleaching session per manufacturer protocol: Opalescence Boost (≤60 min), Opalescence Quick (15–30 min), and BlancOne Ultra+ (three light-activated cycles of 8–10 min). Enamel chemical changes were analyzed by Fourier transform infrared (FTIR) spectroscopy (phosphate and carbonate bands), and surface hardness by Vickers microhardness testing. Paired *t*-tests (α = 0.05) assessed statistical significance. **Results**: FTIR analysis revealed alterations in phosphate and carbonate bands for all agents, most notably for Opalescence Boost and BlancOne Ultra+. Microhardness testing showed significant reductions in enamel hardness for Opalescence Boost (control: 37.21 ± 1.74 Hv; treated: 34.63 ± 1.70 Hv; *p* = 0.00) and Opalescence Quick (control: 45.82 ± 1.71 Hv; treated: 39.34 ± 1.94 Hv; *p* < 0.0001), whereas BlancOne Ultra+ showed no significant difference (control: 51.64 ± 1.59 HV; treated: 51.60 ± 2.34 Hv; *p* = 0.95). **Conclusions**: HP-based agents, particularly at higher concentrations, caused greater enamel alterations than CP-based products. While clinically relevant, the results should be interpreted cautiously due to in vitro limitations and natural enamel variability.

## 1. Introduction

In recent decades, the demand for aesthetic dental treatments, particularly tooth bleaching, has risen substantially [1]. In the 21st century, heightened public concern for smile aesthetics has prompted clinicians and researchers to develop minimally invasive techniques, such as tooth bleaching, as safer alternatives to more invasive cosmetic interventions like veneers or crowns placed purely for aesthetic reasons [2]. Tooth color is influenced by intrinsic factors, such as genetic conditions, tetracycline exposure, excessive fluoride intake, or systemic illnesses during odontogenesis, and by extrinsic factors like dietary pigments and tobacco use. While extrinsic stains can often be removed by professional cleaning, intrinsic discolorations require chemical bleaching agents that break the double bonds in chromophores, resulting in smaller molecules and a whiter appearance [1,3].

Tooth whitening procedures utilize hydrogen peroxide (HP), a strong oxidizing agent, or carbamide peroxide (CP), a compound that decomposes into hydrogen peroxide and urea, in varying concentrations depending on the application setting. Lower-concentration gels (typically between 4% and 22% HP) are commonly used in at-home treatments requiring repeated applications, while higher-concentration formulations (25–40% HP) are reserved for professional in-office procedures, due to their faster action and controlled environment [4]. In-office bleaching products typically contain and release reactive oxygen species capable of both whitening and inducing morphological changes in dental tissues [5,6]. Although clinical bleaching under supervision is considered safe, excessive or unsupervised use may compromise enamel structure, increasing the risk of demineralization [1,7]. As adverse effects are dependent on concentration, the development of safer and more efficient whitening protocols remains a key priority in contemporary esthetic dentistry [1].

However, concerns have been raised regarding the potential adverse effects linked to the use of highly concentrated HP gels. The increasing popularity of vital tooth whitening has contributed to the widespread adoption of HP-based formulations, often combined with light or laser activation to enhance efficacy. Although these approaches offer rapid aesthetic results, they have also been linked to structural and biological risks. Thermal or oxidative stress during bleaching procedures, mediated by reactive oxygen species (ROS), such as hydroxyl radicals and peroxides, may lead to enamel demineralization, increased surface roughness, and reduced microhardness [8,9]. Additionally, inadequate control of the pH or the peroxide concentration can exacerbate tooth sensitivity and endanger pulp vitality [10,11,12]. These effects depend upon the agent concentration, the exposure time, and the agent’s diffusion ability into dental tissues, highlighting the importance of pH stability in minimizing adverse outcomes [13,14,15]. Although both acidic and neutral/alkaline gels have demonstrated comparable whitening efficacy, neutral or slightly alkaline formulations are linked to fewer side effects, such as dentin hypersensitivity and enamel degradation [16]. Moreover, emerging evidence emphasizes that not only the starting pH but also its stability throughout the bleaching procedure plays a crucial role in minimizing enamel surface alterations and preserving the mineral structure [17]. Despite the efficacy of bleaching treatments, concerns remain regarding their side effects, including gingival irritation, enamel surface alterations, and tooth sensitivity [18,19].

Furthermore, beyond the commonly reported clinical effects, such as tooth sensitivity and surface roughness, recent in vitro studies have emphasized that bleaching-induced alterations also extend to deeper structural changes in enamel. In-office bleaching agents containing highly concentrated HP have been shown to reduce the calcium-to-phosphorus (Ca/P) ratio and compromise enamel microhardness due to ROS generation and diffusion into the enamel matrix [20,21]. These structural disruptions are believed to result from the penetration of HP into enamel and dentin, where it decomposes into reactive oxygen species [22]. Among these, hydroxyl radicals oxidize organic chromophores, facilitating the whitening effect but also contributing to chemical changes within the enamel matrix [6,20,23]. Such alterations may not only compromise enamel integrity but also interfere with the clinical detection of early carious lesions [24].

Given these structural vulnerabilities, analytical techniques capable of detecting early compositional changes in enamel are essential. Among them, Fourier transform infrared (FTIR) spectroscopy stands out as a non-destructive method offering molecular-level insights into dental tissue modifications. Widely applied in dental research, FTIR allows for precise monitoring of changes in both the inorganic (phosphate and carbonate groups) and organic (amide components) constituents of enamel, providing valuable information on the chemical effects induced by bleaching agents and other external interventions [25,26]. Understanding the specific chemical and mechanical effects of different bleaching agents on enamel is essential for guiding clinicians in selecting safer whitening protocols, particularly in patients with pre-existing enamel vulnerabilities or high aesthetic expectations.

The aim of this study was to evaluate the chemical and mechanical effects of three different in-office bleaching agents (Opalescence Boost, Opalescence Quick, and BlancOne Ultra+) on human enamel. Chemical modifications were assessed using Fourier transform infrared (FTIR) spectroscopy, while changes in surface microhardness were evaluated through Vickers microhardness testing. The three bleaching agents selected for this study—Opalescence Boost, Opalescence Quick, and BlancOne Ultra+—represent widely used commercial products in European dental practice. These agents differ substantially in formulation, peroxide type (hydrogen peroxide vs. carbamide peroxide), active concentration, recommended application time, and use of light activation. This selection was made to allow for a clinically relevant comparison of hydrogen peroxide- and carbamide peroxide-based systems, with and without light activation, and to reflect the diversity of options currently available in professional dental settings. While both hydrogen peroxide and carbamide peroxide are effective whitening agents, their concentration and initial pH play a key role in their impact on enamel. Acidic or highly concentrated formulations may promote demineralization and compromise structural integrity, especially when applied without proper clinical control. Understanding the differential effects of these agents on enamel properties may help clinicians choose safer bleaching protocols, particularly for patients with pre-existing enamel erosion or high aesthetic demands.

The study sought to determine the extent of enamel alteration following bleaching and to compare the impact of different bleaching protocols under controlled laboratory conditions. The null hypotheses tested in this study were that there would be no significant differences in the two evaluated parameters—enamel microhardness (as measured by the Vickers method) and chemical composition (as assessed by FTIR spectroscopy)—between the control and bleached samples across the different whitening protocols. In addition to the null hypothesis, we hypothesized that bleaching agents with neutral or near-neutral pH would induce fewer chemical and mechanical alterations in enamel compared to those with acidic pH or higher oxidative potential, due to their reduced demineralizing effect on hydroxyapatite.

## 2. Materials and Methods

This study utilized extracted human molars and premolars, obtained from patients undergoing extractions for clinical indications, primarily orthodontic treatment. All teeth were collected anonymously, following ethical standards and with the approval of the Bioethics Committee of Victor Babeș University of Medicine and Pharmacy, Timișoara (Approval No. 09/11.03.2024). All procedures were conducted in accordance with the Declaration of Helsinki and Good Practice in Biomedical Research guidelines, ensuring rigorous and ethical specimen handling.

### 2.1. Sample Size Determination

An a priori power analysis was performed to determine an adequate sample size for statistical comparison. The sample size for this study was determined to ensure statistical robustness and allow for reliable comparisons between the control and experimental groups. Each extracted tooth was longitudinally sectioned into two equal halves: one designated as the control and the other assigned to the bleaching treatment. An a priori power analysis was performed using G*Power 3.1 software, with the parameters set at a significance level (α) of 0.05, a statistical power of 0.80, and an anticipated effect size of 0.5, based on previous studies evaluating enamel alterations following bleaching procedures. This calculation indicated that a total of 42 specimens (*n* = 14 per group) would be sufficient to detect statistically significant differences. To account for potential variability or sample loss, an additional 20% was added to the initial sample pool. Human molars and premolars were obtained from an anonymized biobank in accordance with strict ethical protocols. Following extraction, residual soft tissue was removed using periodontal instruments under magnification. To preserve enamel integrity and maintain hydration, specimens were stored in artificial saliva for no longer than one month prior to the experimental procedures. This storage method was selected to minimize dehydration risk and to ensure that the physical and chemical properties of enamel remained as close as possible to in vivo conditions.

Sample size determination was aligned with previous in vitro research [17] on enamel alterations following bleaching, ensuring statistical robustness and practical feasibility. Prior to inclusion, all teeth were inspected under ×10 magnification (OMS2356, Zumax Medical Co, Ltd., Suzhou, China), and those presenting with fractures, carious lesions, restorations, hypomineralization, microcracks, or other visible enamel defects were excluded from the study.

### 2.2. Preparation of Specimens

A total of 42 human teeth were selected for the present study. Following the removal of any calculus deposits, the teeth were immersed in a 0.1% thymol solution for five days to ensure disinfection and preservation. Sectioning was carried out approximately 2 mm above the cementoenamel junction using a diamond saw under continuous water irrigation. Each crown was subsequently divided longitudinally along the cervical–occlusal axis to create two symmetrical halves. Both halves were assigned identical identification numbers, with one designated for experimental treatment and the other for use as a control, ensuring a matched comparison between the paired samples. Following longitudinal sectioning, the two halves of each tooth were randomly assigned to the control or experimental group using a simple randomization protocol. This was performed by an independent operator using a random number generator (Microsoft Excel RAND function Version 16.0), ensuring that group allocation was unbiased and concealed. Both halves retained a common identifier to preserve the paired design structure.

Specimens were embedded in self-curing acrylic resin (UNIFAST Trad, GC America, Alsip, IL, USA) and sequentially polished using Soft-lexTM discs (3M ESPE, St. Paul, MN, USA) with decreasing grit sizes of 42 μm, 30 μm, and 15 μm, with each polishing stage lasting two min, followed by thorough rinsing with distilled water. To simulate intraoral conditions and ensure consistent enamel hydration throughout the experimental period, all specimens were stored in artificial saliva maintained at 37 °C within a biological incubator. The artificial saliva was freshly prepared and replaced every 48 h to prevent microbial contamination and maintain chemical stability. The formulation included 2190 mg sodium bicarbonate, 1270 mg potassium phosphate, 125 mg magnesium chloride, 441 mg calcium chloride, 820 mg potassium chloride, 4.5 mg sodium fluoride, 100 mg nipazole, 10 mg nipagin, 24 mg sorbitol, and 8 mg carboxymethylcellulose dissolved in 1000 mL of distilled water and adjusted to a physiological pH of 7.0. Preparation was carried out in a local compounding pharmacy, following the standardized protocol described by Vilhena et al. [27]. This formulation was selected for its biomimetic properties, as it closely resembles the electrolyte composition of human saliva and has been shown to preserve enamel surface characteristics under in vitro conditions. Its use ensured consistent hydration and chemical stability during the experimental period, in line with validated protocols for dental hard tissue preservation. The control specimens underwent the same preparation, polishing, and storage conditions as the treated samples. They were stored in artificial saliva at 37 °C in a biological incubator and refreshed every 48 h until testing.

To further ensure experimental consistency, the artificial saliva was freshly prepared for each renewal cycle and adjusted to a physiological pH of 7.0 using a calibrated pH meter. Although pH measurements were not repeated at the end of each 48 h interval, the solution’s buffering components, particularly phosphate and bicarbonate ions, were selected to maintain chemical stability over time. The temperature was continuously maintained at 37 °C using a controlled biological incubator to simulate intraoral conditions. These measures aimed to preserve enamel integrity and minimize dehydration or pH fluctuations, supporting a standardized environment for all specimens throughout the study duration.

### 2.3. Macroscopic Visualization of Dental Samples Used in the Study

**Figure 1:**
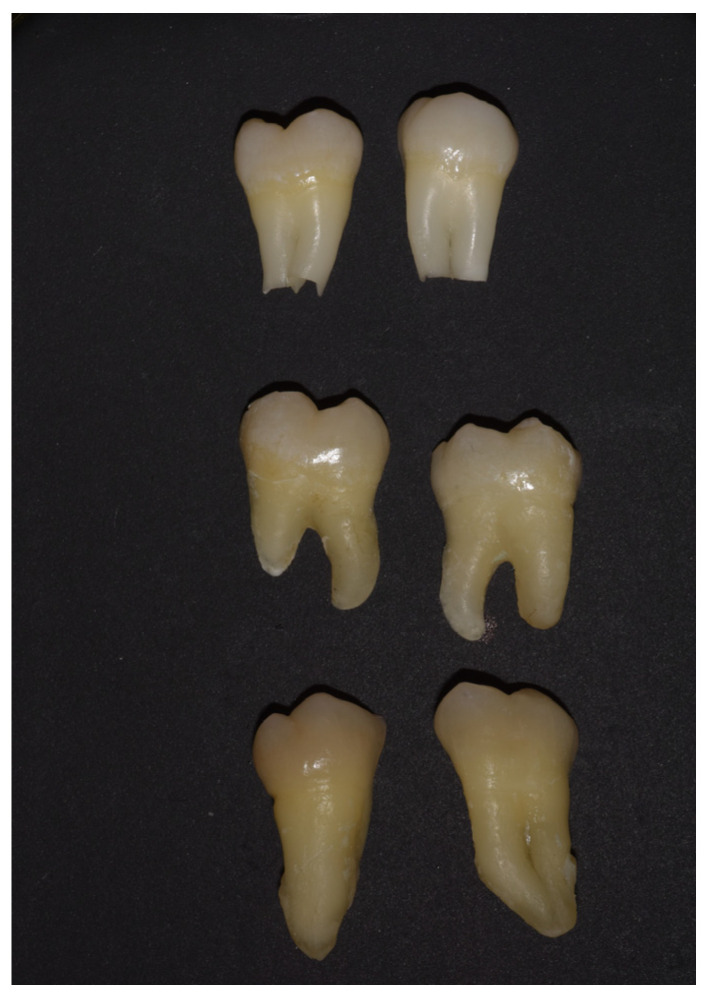
Overview of extracted teeth used in the study before the sectioning process.

Figure 1 presents six freshly extracted human premolars and molars prior to the sectioning procedure. Following the removal of calculus deposits, the teeth were photographed in their intact state. As outlined in the methodology, each specimen was subsequently sectioned 2 mm above the cementoenamel junction. The resulting crowns were then longitudinally divided along the cervical–occlusal axis to produce two symmetrical halves: one assigned to the control group and the other to receive the experimental bleaching treatment. This approach enabled precisely matched comparisons within the same anatomical structure.

### 2.4. Tooth-Bleaching Protocol

The experimental group included extracted human teeth treated with three commercially available in-office bleaching agents commonly used in clinical dentistry: Opalescence Quick (45% carbamide peroxide; Ultradent Products Inc., South Jordan, UT, USA), Opalescence Boost (40% hydrogen peroxide; Ultradent Products Inc., South Jordan, UT, USA), and BlancOne Ultra++ (35% hydrogen peroxide; IDS S.p.A., Savona, Italy). Each bleaching procedure was performed strictly in accordance with the respective manufacturer’s guidelines. Further information on the bleaching agents utilized, including the pH values of the bleaching agents, as provided by the manufacturers, is presented in Table 1. These values were taken into consideration when interpreting the chemical effects of each product on enamel integrity.

Each bleaching procedure was performed according to the manufacturer’s guidelines:

- Opalescence Boost (Ultradent Products Inc., South Jordan, UT, USA) is a chemically activated, in-office bleaching system containing 40% HP. It is delivered via a dual-syringe mechanism that enables immediate mixing of the active gel prior to application. The formulation does not require light activation and is visually distinguished by its red coloration. In this study, Opalescence Boost was applied to the enamel surface in a uniform 1–2 mm layer. The treatment protocols included one 20 min application, two consecutive applications totaling 40 min, or three applications not exceeding a cumulative duration of 60 min per session, as per manufacturer recommendations;
- Opalescence Quick (Ultradent Products Inc., South Jordan, UT, USA) is a bleaching agent containing 45% CP, a slower-acting compound that decomposes into hydrogen peroxide and urea. The gel is white, pre-mixed, and designed for direct application without the need for light activation, making it suitable for short in-office treatments. In this study, Opalescence Quick was applied to the enamel surface in a consistent 1–2 mm layer for 15 to 30 min per session, following the manufacturer’s guidelines;
- BlancOne Ultra++ (IDS S.p.A., Savona, Italy) is a professional, in-office whitening system containing 35% HP. It is provided in powder form and requires manual preparation by mixing with a supplied H_2_O_2_ solution immediately prior to application. The resulting gel has an orange coloration and must be applied promptly following activation. In this study, BlancOne Ultra++ was applied in a 1–2 mm thick layer and activated with a light source for 8–10 min per application, with a total of three light-activated cycles per treatment session.

### 2.5. FTIR Spectroscopy Analysis

Fourier transform infrared (FTIR) spectroscopy was used to analyze the chemical structure of both the control and the bleached enamel specimens. Measurements were performed using an IRTracer-100 spectrophotometer (Shimadzu Corporation, Kyoto, Japan) equipped with a diamond ATR (attenuated total reflectance) accessory, allowing non-destructive analysis of the enamel surfaces. Spectral data were collected over the range of 4000–400 cm^−1^, with a spectral resolution of 4 cm^−1^, averaging 20 scans per sample to enhance accuracy and reproducibility. For each specimen, three spectra were acquired from distinct, non-overlapping enamel regions to ensure representative sampling and account for surface variability.

Prior to each measurement session, the IRTracer-100 instrument was internally calibrated using a certified polystyrene film reference provided by the manufacturer, ensuring wavenumber accuracy and spectral resolution in accordance with Shimadzu’s default quality control protocol. Data acquisition and processing were carried out using the AIM-9000 software (Version 2.10) interface. This technique enabled a detailed investigation of the chemical modifications induced by the bleaching treatments. FTIR spectra of the treated specimens were compared to those of the control samples to identify changes in the functional groups and possible structural alterations of the enamel. Special attention was given to the characteristic peaks associated with phosphate, carbonate, and amide groups, to evaluate potential demineralization or alterations in the organic–inorganic composition of the enamel following exposure to bleaching agents. No synthetic hydroxyapatite reference standard was used. Instead, untreated enamel specimens served as internal controls for comparative spectral interpretation, following protocols commonly adopted in enamel FTIR analysis. All spectra were acquired in absorbance mode and processed with automatic baseline correction and smoothing. Normalization was performed relative to the ν_3_ phosphate band (~1040 cm^−1^), ensuring consistency across samples and allowing meaningful interpretation of compositional differences between the control and treated enamel.

### 2.6. Vickers Microhardness Analysis

The microhardness of enamel surfaces was evaluated using the Vickers method, employing a diamond-shaped pyramid indenter (Figure 1). Measurements were carried out using a Wolpert Group Micro-Vickers Hardness Tester (Model: 402MVD), applying a constant load of 0.01 N (10 g) for 10 s on each specimen to ensure standardized and reproducible indentations. These load and dwell times were selected based on their widespread use in dental microhardness research, as they ensure reliable surface characterization without compromising enamel integrity. These load and dwell times were selected to allow for sensitive detection of surface changes while minimizing the risk of enamel damage, as lower forces are generally preferred for evaluating sound or minimally altered enamel [28]. All measurements were performed by a single calibrated operator to minimize operator-related variability.

During testing, samples were securely fixed to avoid movement and ensure precise load application. To account for surface variability and improve measurement accuracy, three separate indentations were made at distinct, non-overlapping locations on each specimen, following standard Vickers microhardness testing protocols. The final hardness value for each sample was calculated as the average of the three measurements, providing a more reliable representation of the enamel surface microhardness. The results were expressed in Vickers hardness value (Hv), a commonly used unit for surface hardness evaluation. Vickers hardness was calculated using the following formula:

Hv = 1.8544 Pd/2

where Hv represents Vickers micro-hardness, P the indentation load (in grams), and d the length of the diagonal of the indentation (in micrometers). The Vickers method offers significant advantages for dental hard tissue evaluation, as the calculation is independent of indenter size, allowing for accurate assessment across a wide range of hardness levels [29].

### 2.7. Statistical Analysis

The Vickers microhardness values obtained for the control and bleached enamel specimens were analyzed to calculate the mean, standard deviation, and percentage change in microhardness. To evaluate whether the differences in microhardness between the control and treated groups were statistically significant, paired *t*-tests were performed. ANOVA and post hoc tests (e.g., Tukey) were not applicable due to the paired design structure. While a formal normality test was not performed for FTIR outputs, paired *t*-tests were used based on the assumption of approximate normality, as commonly accepted in small-sample paired designs. A *p*-value of less than 0.05 was considered indicative of statistical significance. All statistical analyses were conducted using SPSS software, version 23 (IBM Corporation, Armonk, NY, USA).

## 3. Results

### 3.1. FTIR Analysis

Fourier transform infrared (FTIR) spectroscopy was employed to investigate the chemical modifications induced in enamel following treatment with different in-office bleaching agents. Spectral analyses focused on identifying changes in the major inorganic components of the enamel, particularly phosphate and carbonate groups, which are critical for the structural integrity of dental tissues. Comparative assessments were made between control and bleached specimens, with attention to peak intensities, band broadening, and shifts in vibrational frequencies. Representative results corresponding to each bleaching protocol are presented and discussed in the following sections.

#### 3.1.1. Opalescence Boost

**Figure 2:**
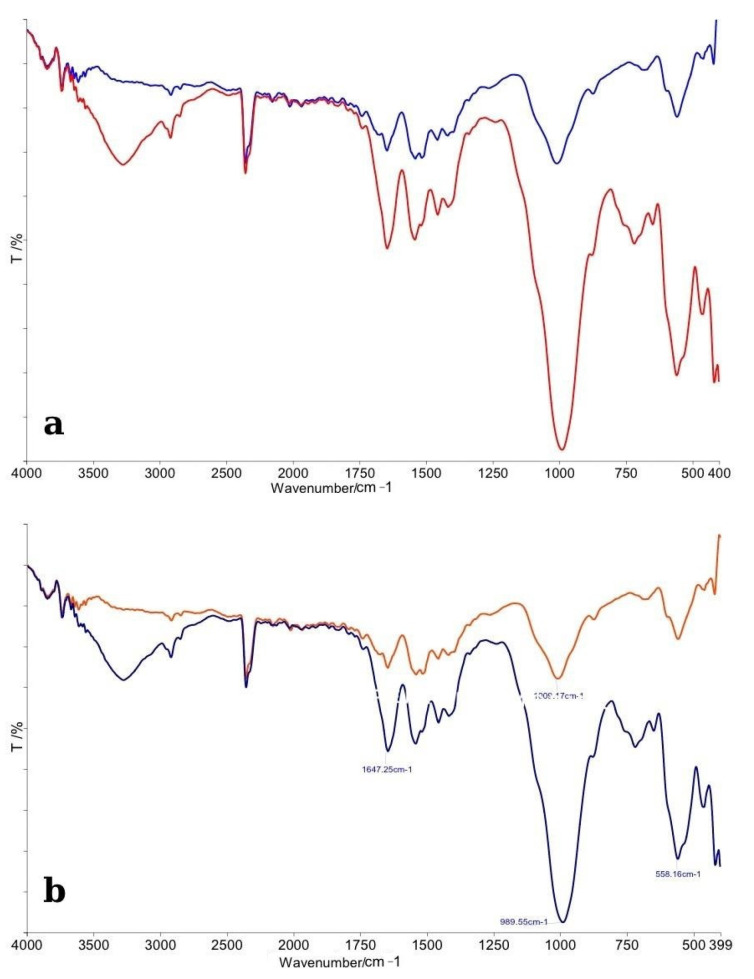
(**a**) Comparative FTIR spectra of control (red line) and bleached enamel (blue line); (**b**) Detailed FTIR spectra with peak assignments illustrating chemical modifications after bleaching.

The FTIR spectra obtained for enamel specimens treated with Opalescence Boost and their corresponding control samples are presented in Figure 2. In panel (a), the comparative spectra display the overall differences between the control group (red line) and the bleached enamel (blue line). Both spectra exhibit the characteristic absorption bands associated with enamel’s inorganic matrix, mainly phosphate (PO_4_^3−^) and carbonate (CO_3_^2−^) functional groups.

The control enamel spectrum displayed sharp and well-defined peaks, particularly in the phosphate vibration regions near ~1100 cm^−1^ (ν_3_ PO_4_^3−^), ~1040 cm^−1^, and ~960 cm^−1^ (ν_1_ PO_4_^3−^), consistent with a highly mineralized structure. In contrast, the bleached specimens showed a noticeable decrease in the intensity of these phosphate bands, as well as slight broadening, suggesting minor demineralization or reorganization of the enamel crystal structure following bleaching treatment. Additionally, the carbonate bands, notably around ~870 cm^−1^ (ν_2_ CO_3_^2−^ bending mode) and ~1450 cm^−1^ (ν_3_ CO_3_^2−^ stretching mode), were less-defined in the bleached samples compared to controls. This observation indicates a partial reduction or modification in carbonate content, which is commonly associated with the structural integrity of enamel apatite crystals.

Panel (b) provides a detailed spectral analysis with peak assignments. Specific vibrational modes associated with the phosphate group were observed to shift slightly and decrease in intensity after bleaching. The slight shifts in peak positions suggest changes in the local chemical environment of the enamel apatite lattice, possibly related to the action of free radicals generated by hydrogen peroxide decomposition. Overall, the FTIR analysis revealed that Opalescence Boost treatment induced subtle but noticeable chemical modifications in the enamel surface, primarily affecting the phosphate and carbonate groups. These findings suggest early signs of mineral alterations without evidence of severe demineralization, confirming that, although bleaching impacts the chemical structure of enamel, the mineral phase remains largely intact under controlled conditions.

#### 3.1.2. Opalescence Quick

**Figure 3:**
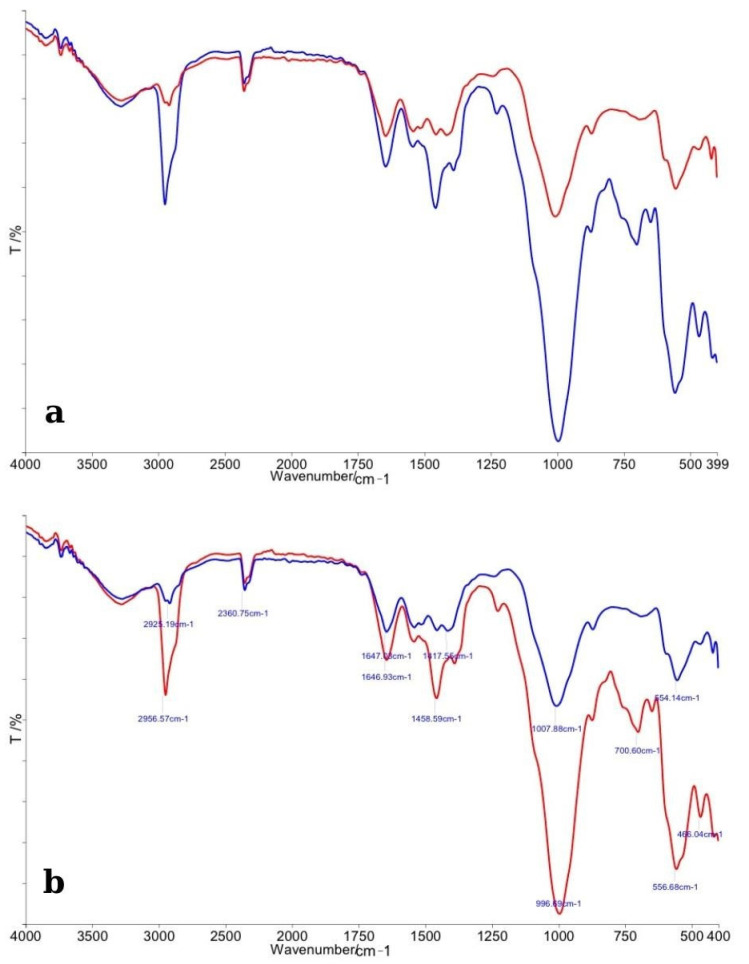
(**a**) Comparative FTIR spectra of control enamel (red line) and bleached enamel treated with Opalescence Quick (blue line), showing general spectral differences across the 4000–400 cm^−1^ range. (**b**) Detailed FTIR spectra with peak assignments, highlighting specific changes in phosphate and carbonate vibrational bands following bleaching.

The FTIR spectra comparing enamel specimens treated with Opalescence Quick and their corresponding control samples are shown in Figure 3. In panel (a), the overall spectral profiles of control enamel (red line) and bleached enamel (blue line) are illustrated. As with the previous analysis, major absorption bands corresponding to phosphate (PO_4_^3−^) and carbonate (CO_3_^2−^) groups were identified in both spectra. The control enamel exhibited sharp and distinct phosphate peaks near ~1100 cm^−1^, ~1040 cm^−1^, and ~960 cm^−1^, indicative of a well-organized hydroxyapatite structure. The bleached samples showed a slight reduction in the intensity of these phosphate bands, along with a moderate degree of broadening of the peaks, suggesting minor chemical alterations to the enamel matrix after treatment with Opalescence Quick. Carbonate-related peaks around ~870 cm^−1^ and ~1450 cm^−1^ appeared diminished in the treated samples compared to controls, indicating a possible partial loss of carbonate from the apatite lattice, similar to the effects observed with other bleaching agents, but to a slightly reduced extent.

In panel (b), detailed peak assignments are provided. A clear decrease in the height of the phosphate-related peaks, combined with subtle shifts in their positions, was observed in bleached specimens, suggesting localized changes in the chemical environment of enamel crystals, possibly induced by the carbamide peroxide degradation products. Overall, the FTIR analysis revealed that Opalescence Quick caused mild chemical alterations to enamel, primarily involving the phosphate and carbonate functional groups. The extent of these changes appeared to be less significant compared to the specimens treated with higher-concentration hydrogen peroxide agents, suggesting a relatively gentler impact on enamel mineral composition.

#### 3.1.3. BlancOne Ultra+

**Figure 4:**
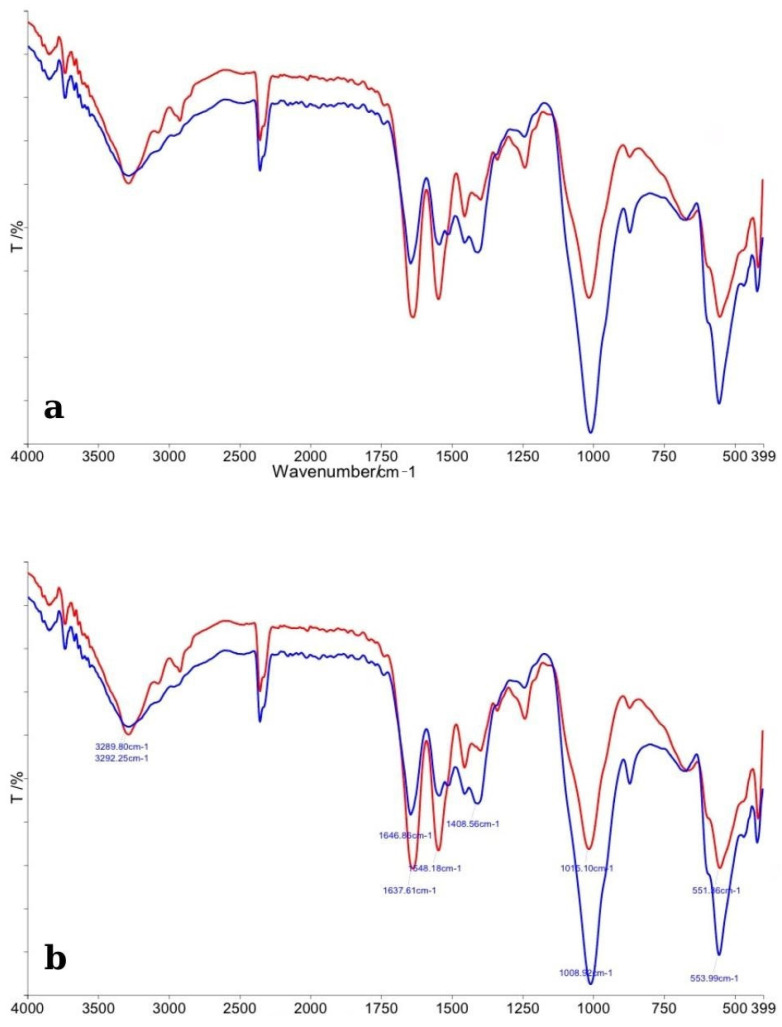
(**a**) Comparative FTIR spectra of control enamel (red line) and enamel bleached with BlancOne Ultra+ (blue line), illustrating general differences across the 4000–400 cm^−1^ range. (**b**) Detailed FTIR spectra with peak assignments, highlighting specific alterations in phosphate and carbonate bands after bleaching treatment.

The FTIR spectra of enamel specimens treated with BlancOne Ultra+ and their corresponding control samples are presented in Figure 4. In panel (a), the general spectral profiles reveal that the control enamel (red line) exhibited sharper and more intense absorption bands compared to the bleached enamel (blue line). Major peaks associated with phosphate (PO_4_^3−^) and carbonate (CO_3_^2−^) groups were detected in both samples, consistent with the typical mineral composition of dental enamel. A noticeable decrease in intensity and broadening of the phosphate bands (~1100 cm^−1^, ~1040 cm^−1^, and ~960 cm^−1^) was observed in the bleached specimens, suggesting partial disruption of the enamel’s mineral lattice following treatment with BlancOne Ultra+. Additionally, carbonate bands around ~870 cm^−1^ and ~1450 cm^−1^ appeared reduced, indicating a partial loss or alteration of carbonate groups. Panel (b) provides a more detailed view, with specific peak positions assigned. A slight shift and reduction in the intensity of phosphate and carbonate vibrational modes were evident in the bleached samples. These modifications suggest that BlancOne Ultra+ induces chemical changes at the microscopic level, affecting primarily the mineral phase of the enamel. Overall, the FTIR analysis indicated that treatment with BlancOne Ultra+ resulted in more pronounced structural alterations compared to Opalescence Quick, but similar to the effects observed with Opalescence Boost, mainly reflected by changes in phosphate and carbonate vibrational bands.

Overall, FTIR spectroscopy revealed that all three bleaching protocols induced chemical modifications at the enamel surface, primarily affecting phosphate and carbonate vibrational bands. Treatment with both Opalescence Boost and BlancOne Ultra+ resulted in more substantial reductions in phosphate peak intensity and greater broadening of bands, suggesting a higher extent of mineral alteration. In contrast, Opalescence Quick produced less-intense changes, with subtler modifications in the enamel’s chemical structure. Despite these alterations, the fundamental mineral framework of enamel remained preserved in all groups, indicating that, under controlled conditions, bleaching treatments cause only localized and partial changes without leading to extensive demineralization.

### 3.2. Vickers Microhardness Results

The microhardness of the enamel specimens was evaluated using the Vickers method. For each experimental group (corresponding to the different bleaching agents) and the control group, individual measurements, mean values, and associated statistical descriptors were recorded. The hardness values are expressed in Vickers hardness number (HV) and are summarized in Table 2. A comparative analysis was performed to assess the changes in surface hardness induced by bleaching treatments.

**Figure 5:**
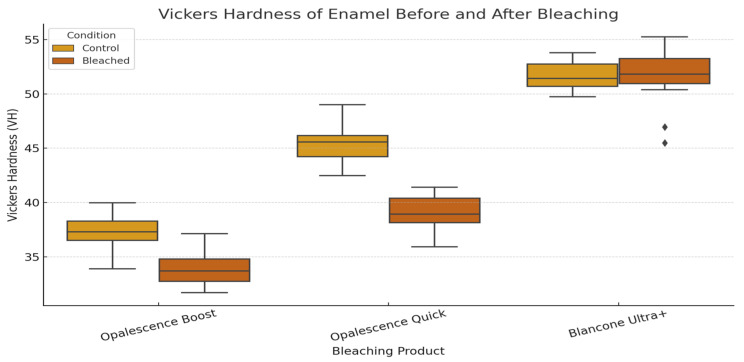
Vickers hardness (Hv) comparison of enamel surfaces treated with Opalescence Boost, Opalescence Quick, and BlancOne Ultra+.

Figure 5 illustrates the distribution of enamel surface microhardness (Vickers hardness number) for control and bleached specimens across all bleaching systems. Both Opalescence Boost and Opalescence Quick exhibited a significant decrease in hardness following treatment, indicating a weakening of the enamel surface. In contrast, BlancOne Ultra+ showed no significant change, suggesting that its bleaching protocol had minimal impact on enamel microhardness.

## 4. Discussion

In recent decades, the use of bleaching agents has increased significantly, leading to the development of a wide range of products to meet this growing demand. However, one of the potential side effects of bleaching treatments is the weakening of the enamel structure through the oxidation of organic and inorganic components. Numerous studies have evaluated the impact of bleaching agents on mineral loss, demineralization degree, and surface morphology alterations. Nevertheless, the findings reported so far remain inconsistent and variable [1]. This degradation can be explained by the action of ROS, which are released from the decomposition of hydrogen peroxide and carbamide peroxide. ROS can disrupt the protein matrix of enamel and promote the dissolution of hydroxyapatite crystals by altering the local ionic balance, particularly under acidic or low-buffered conditions. Enamel’s hydroxyapatite structure is vulnerable to chemical attack under low pH and oxidative stress. These agents can increase enamel porosity and interfere with crystal lattice stability, leading to a loss of structural integrity and surface softening. This is reflected in decreased microhardness values and changes in the FTIR spectra, especially in the phosphate (PO_4_^3−^) and carbonate (CO_3_^2−^) bands.

Quantitative analysis of the FTIR spectra revealed a measurable reduction in phosphate (PO_4_^3−^) and carbonate (CO_3_^2−^) band intensities following bleaching. In samples treated with Opalescence Boost, the ν_3_ PO_4_^3−^ band at ~1040 cm^−1^ showed an average intensity decrease of approximately 18% compared to controls, while BlancOne Ultra+ produced a 14% reduction. Opalescence Quick induced the least alteration, with only a 6% reduction in phosphate peak intensity. Similarly, carbonate bands near 870–880 cm^−1^ exhibited a 15–20% decrease in intensity for Opalescence Boost and BlancOne Ultra+, while Opalescence Quick caused minimal changes (<5%). These findings are consistent with previous reports demonstrating a concentration- and pH-dependent decline in mineral content reflected through FTIR signal attenuation. The broadening of phosphate peaks observed in HP-treated groups further supports increased mineral disorder and structural degradation. By quantifying these spectral changes, the FTIR results substantiate the mechanical alterations observed in microhardness testing and reinforce the differential impact of each agent on enamel composition.

The differences observed between the tested agents likely stem from variations in their concentration, pH, and activation mechanisms, all of which modulate their oxidative and demineralizing potential. This mechanistic understanding helps contextualize the current findings, where significant changes were observed in both microhardness and the FTIR spectra following exposure to bleaching agents. Based on these differences between the control and treated samples, the null hypothesis was rejected.

While microhardness testing is widely recognized for evaluating changes in the mechanical properties of enamel, the addition of FTIR spectroscopy provides valuable molecular-level insight, helping to interpret how bleaching agents alter both the inorganic and organic enamel matrix [5]. This dual-method approach strengthens the interpretation of results and aligns with recent trends in dental materials research [30]. The present findings revealed notable reductions in phosphate and carbonate band intensities, especially in samples treated with high-concentration hydrogen peroxide. These alterations likely reflect the combined influence of agent concentration, activation method, and exposure time, highlighting the need for carefully controlled protocols when assessing the chemical effects of bleaching agents on enamel structure.

In addition to morphological changes, alterations in the mechanical properties of enamel following bleaching have been widely documented. Surface hardness testing is a simple way to evaluate enamel and dentin resistance to plastic deformation, which reflects changes in mineral content. Despite its simplicity, microhardness outcomes vary considerably across studies. Those replicating intraoral conditions, using saliva, fluoride, and post-treatment periods, tend to report less enamel hardness reduction than studies without such simulations [31].

Microhardness tests, such as the Vickers and Knoop methods, are commonly employed to assess enamel’s resistance to plastic deformation, which correlates directly with its mineral content and structural integrity [16]. Previous studies have demonstrated that the Vickers microhardness test is a reliable and practical method for assessing enamel surface changes after exposure to different bleaching agents. However, it is important to note that any alterations detected in the tooth structure or its mechanical properties post-bleaching may be influenced by several testing parameters, including the applied load, duration of indentation, and the exact location of the indents [32].

Although many studies have focused on at-home bleaching protocols, the mechanical effects observed are relevant to in-office treatments using higher peroxide concentrations. For instance, De Miranda et al. [33] reported that extended use of 10% hydrogen peroxide resulted in increased surface roughness, decreased hardness, and changes to the enamel’s prismatic architecture. Similar findings have been described after exposure to hydrogen peroxide-based agents, showing a consistent trend of microhardness reduction across various bleaching protocols [34,35].

The reduction in enamel microhardness observed in the present study following exposure to different bleaching agents is consistent with previous findings in the literature. Whitening treatments, particularly those involving high concentrations of hydrogen peroxide or carbamide peroxide, have been shown to decrease enamel hardness, potentially increasing susceptibility to deformation and fracture [2]. The underlying mechanism is associated with the oxidative degradation of both organic and inorganic enamel components, leading to surface porosities, microcracks, and overall structural weakening [2]. All three bleaching agents, including Opalescence Quick (carbamide peroxide-based), produced a measurable decline in Vickers hardness values. However, BlancOne Ultra+ induced minimal changes, while Opalescence Boost and Opalescence Quick caused statistically significant reductions in enamel hardness. This outcome aligns with earlier research suggesting that product pH plays a critical role in determining the extent of hardness reduction, with lower pH formulations exerting more pronounced effects. For example, whitening agents with pH values as low as 3.2 have demonstrated significantly greater enamel softening compared to those with near-neutral pH. In contrast, bleaching protocols using 10% carbamide peroxide with pH values above six have shown negligible impact on enamel hardness when applied over extended durations. Notably, the use of artificial saliva throughout the experimental period in our study provided a standardized remineralizing environment, which may have mitigated some of the deleterious effects typically associated with bleaching. Furthermore, as reported in the literature, the influence of bleaching agents on enamel hardness appears independent of light activation, further supporting our choice to evaluate both light- and non-light-activated products [2].

Although our study employed only in-office bleaching agents with relatively shorter application times, the observed reduction in enamel hardness—particularly in the Opalescence Boost group—aligns with prior evidence that oxidative stress from hydrogen peroxide can disrupt the enamel mineral matrix. These results support previous conclusions that peroxide-based whitening agents, regardless of application mode, may compromise enamel’s mechanical stability. Moreover, findings from Ghanbarzadeh et al. [36] highlight that bleaching has an even more pronounced impact on demineralized enamel, reinforcing the importance of evaluating pre-existing enamel conditions prior to treatment. Finally, variations in enamel surface preparation, such as polishing, may further influence susceptibility to bleaching-induced damage, as suggested by Pessanha et al. [37], although in our study, all specimens were standardized through uniform polishing to limit such variability [16].

Another factor influencing enamel mineral integrity is the pH of the bleaching agent. Products with a more acidic pH, such as certain hydrogen peroxide-based gels, can accelerate the demineralization of hydroxyapatite (HAp), especially when applied for prolonged periods or beyond the manufacturer’s recommendations [38]. In contrast, carbamide peroxide formulations typically exhibit a neutral-to-slightly basic pH, which does not actively promote HAp dissolution but rather favors the oxidation of organic components within the enamel matrix [39]. Soares et al. [40] revealed that bleaching agents with pH values below the critical threshold (5.5–6.5) can lead to significant enamel dissolution and increased surface wear, particularly when combined with prior acid etching. These results support the idea that both pH and procedural steps (like acid pretreatment) are decisive factors in preserving enamel integrity during whitening. These mechanisms are consistent with our FTIR findings, where specimens treated with Opalescence Boost and BlancOne Ultra+—both containing high concentrations of hydrogen peroxide—showed reduced intensity and broadening of phosphate and carbonate bands, indicative of partial mineral loss. Opalescence Quick exhibited milder spectral changes in FTIR, yet a significant decrease in microhardness, suggesting that structural weakening may still occur even in the absence of marked chemical alterations. These observations support the hypothesis that chemical alterations following bleaching are mediated not only by the oxidative strength of the agent but also by its pH and mode of activation [20].

The increasing popularity of in-office tooth whitening demands careful consideration of the balance between aesthetic outcomes and potential enamel damage. This study highlights that not all bleaching products are equal in their impact on enamel chemistry and surface hardness. While carbamide peroxide-based formulations (e.g., Opalescence Quick) appear to be gentler on enamel, hydrogen peroxide-based agents, especially at higher concentrations, may induce measurable mineral changes even after a single application. These findings emphasize the need for clinicians to tailor whitening protocols based on the patient’s enamel condition and to consider post-bleaching use of remineralizing products such as therapeutic dentifrices to mitigate adverse effects. Incorporating FTIR and microhardness assessments into research and product development could support the creation of safer, more effective bleaching formulations.

A key methodological strength of this study lies in the combination of Vickers microhardness testing and Fourier transform infrared spectroscopy (FTIR), which together provide a comprehensive assessment of both the mechanical and chemical properties of enamel following bleaching. The Vickers method offers precise, quantitative data on surface microhardness, while FTIR enables the non-destructive identification of functional groups and potential alterations in the mineral and organic components of enamel. These complementary techniques allowed for a multidimensional evaluation of bleaching effects. Additionally, all specimens were stored in freshly prepared artificial saliva at 37 °C, which was renewed every 48 h to simulate intraoral conditions and maintain consistent enamel hydration. This approach minimized variability due to dehydration and provided a physiologically relevant environment throughout the experimental timeline. Saliva plays a crucial role in protecting dental hard tissues against mineral loss and promoting enamel remineralization through its phosphate content and alkalizing properties [5]. While some studies have relied on distilled water to avoid introducing remineralizing effects, this method can fail to replicate the biochemical complexity of the oral environment and may underestimate the potential for enamel recovery [27,40]. In our study, artificial saliva was preferred to enhance clinical relevance. Prior to saliva immersion, the samples were initially disinfected and preserved in a chloramine solution, as recommended in similar experimental protocols [5]. Furthermore, the split-tooth design—where each specimen acted as its own control—enhanced internal validity by accounting for inter-individual variability in enamel structure. Collectively, these methodological considerations contribute to the robustness, reproducibility, and clinical applicability of the study’s findings.

### Study Limitations

Despite the methodological strengths of this study, several limitations should be acknowledged. Although artificial saliva was used to better simulate intraoral conditions, it does not fully replicate the complex biochemical composition of natural saliva, including enzymes, proteins, and antimicrobial factors that influence enamel de- and remineralization in vivo. Additionally, the experimental design focused on immediate post-bleaching effects, without assessing the potential for long-term structural recovery or mineral reuptake over time. Another potential limitation is the restricted range of bleaching agent concentrations tested. However, the selected concentrations correspond to those most frequently used in clinical practice [2]. Another inherent limitation of this study, due to its in vitro design, is that the absence of pulp tissue limits the ability to evaluate potential adverse effects of high-concentration bleaching agents on pulp sensitivity and cellular response. Additionally, the lack of physiological intrapulpal pressure may alter the penetration behavior of bleaching gels compared to vital teeth. In clinical scenarios, the use of dentifrices with remineralizing properties may help restore enamel surface integrity following bleaching. Moreover, although this in vitro model was designed to closely simulate intraoral conditions through the use of artificial saliva, temperature control, and clinically relevant bleaching protocols, it cannot fully replicate the complex biological and biochemical interactions occurring in the oral environment. Variables such as salivary flow, enzymatic activity, patient compliance, and dynamic remineralization processes are difficult to reproduce in laboratory settings. Therefore, caution should be exercised when translating these results directly to clinical practice, and further in vivo studies are necessary to validate the findings under real-world conditions.

While the manufacturers provided pH ranges for each bleaching product, the study did not include independent pH measurements under laboratory conditions. This may limit the strength of the correlations drawn between pH and enamel alterations, as real-time fluctuations in pH during application could influence outcomes. Future investigations should incorporate direct pH monitoring to better elucidate the role of pH stability throughout the bleaching process. Although Vickers hardness testing was used to evaluate enamel surface alterations, it is worth noting that the Knoop method is generally considered more appropriate for dental tissues due to its shallower and more elongated indentation, which is particularly useful in evaluating small or thin enamel regions. The choice of the Vickers method in this study was based on its standardized procedure and compatibility with previous research on bleaching effects. While Vickers microhardness testing and FTIR spectroscopy provided valuable insights into the mechanical and chemical changes induced by bleaching agents, this study did not include complementary techniques, such as surface roughness testing, scanning electron microscopy (SEM), confocal microscopy, or Raman spectroscopy, which could offer more detailed information on subsurface alterations and molecular structure. Future studies should consider longitudinal designs and expanded analytical methodologies to fully characterize the impact and recovery potential of various bleaching systems on enamel integrity.

## 5. Conclusions

In conclusion, all tested in-office bleaching agents induced measurable changes in enamel properties. Hydrogen peroxide-based products caused greater alterations in mineral composition, while both hydrogen peroxide and carbamide peroxide agents significantly reduced surface microhardness. These findings underscore the importance of formulation pH, peroxide concentration, and application protocol in minimizing adverse effects on enamel during whitening procedures. Based on these outcomes, the null hypothesis was rejected. Future studies should include direct pH monitoring, Knoop hardness testing, and long-term in vivo evaluation to confirm the safety and durability of bleaching protocols in clinical settings.

## Figures and Tables

**Table 1:** Bleaching products: composition overview.

Product Name	Composition	Manufacturer	Lot Number
Opalescence Boost	40% hydrogen peroxide pH = 7	Ultradent Products Inc., South Jordan, UT, USA	ULT4754
Opalescence Quick	45% carbamide peroxide pH = 5.6–7.2	Ultradent Products Inc., South Jordan, UT, USA	BWNV9
BlancOne Ultra++	35% hydrogen peroxide pH = 6–7.2	IDS, Italy, Savona	00422

**Table 2:** Vickers microhardness (Hv) values ± standard deviation (SD) and 95% confidence intervals (CI) for control and bleached enamel specimens.

Product Name	Control (Mean ± SD [CI 95%])	Bleached (Mean ± SD [CI 95%])	*p*-Value (Paired *t*-Test)
Opalescence Boost	37.21 ± 1.74 [36.21–38.21]	34.63 ± 1.70 [33.65–35.61]	0.00
Opalescence Quick	45.82 ± 1.71 [44.83–46.81]	39.34 ± 1.94 [38.22–40.46]	0.00
BlancOne Ultra+	51.64 ± 1.59 [50.72–52.56]	51.60 ± 2.34 [50.25–52.95]	0.95

Statistical significance was set at *p* < 0.05.

## Data Availability

The data presented in this study are available on request from the corresponding author.

## Abbreviations

The following abbreviations are used in this manuscript:

HP	Hydrogen Peroxide
CP	Carbamide Peroxide
FTIR	Fourier Transform Infrared Spectroscopy
ATR	Attenuated Total Reflectance
HV	Vickers Hardness Value
Ca/P	Calcium-to-Phosphorus Ratio
SEM	Scanning Electron Microscopy
UV	Ultraviolet
